# A very rare case of chylomicronemia revealed by cerebral thrombophlebitis in a 4-month-old infant

**DOI:** 10.1016/j.amsu.2022.103276

**Published:** 2022-02-09

**Authors:** Onci-Es-Saad, Oussama Lamzouri, Amine Bouchlarhem, Leila Haddar, Aabdi Mohammed, Hamza mimouni, Kaoutar zerouati, Houssam Bkiyar, Brahim Housni

**Affiliations:** a Faculty of Medicine and Pharmacy, Mohammed I^st^ University, Mohammed First University, Faculty of Medicine and Pharmacy, LAMCESM, Oujda, Morocco; b Department of Anesthesiology and Intensive Care Unit, Mohammed VI University Hospital Mohammed I University, Mohammed First University, Faculty of Medicine and Pharmacy, LAMCESM, Oujda, Morocco; c Department of Pediatric, Mohammed VI University Hospital, Mohammed I University, Oujda, Morocco

**Keywords:** ChylomIcronemia, Cerebral venous thrombosis, Lipoprotein electrophoresis

## Abstract

**Introduction:**

Hyperchylomicronemia is a disorder of lipid's metabolism that can present fatal complications such us such venous or arterial thrombosis, pancreatitis, and cardiovascular incidents.

**Case presentation:**

In this report case we report a 4months old patient who was admitted in the emergency room for hypotonia and during the blood sampling we were surprised by the macroscopic latescent aspect of the blood. During the investigations we found that the patient had a fatty cerebral venous thrombosis that revealed hyperchylomicronemia. Furthermore, the patient presented tuberculosis cerebral abscess and stage A pancreatitis and was successfully treated.

**Discussion:**

Primary hypertriglyceridemia results from the accumulation of genes polymorphisms encoding for proteins involved in the triglycerides metabolism but before thinking about primary origin a secondary one should be pushed aside. Biological investigations should test lipoprotein lipase activity that can be absent or reduced to confirm a lipid disorder, then lipoprotein electrophoresis and genetic study can deliver the diagnosis. The management of this disease is based on low fat diet that should not be over than 25–30g per day, also statin, fibrate, omega 3 acid, heparin and insulin can be used.

**Conclsuion:**

Adequate treatment and exploration permits to obtain the optimum care to avoid any complications.

## Highlights

• 4months year old patient admitted for hypotonia and drowsiness.
• The diagnosis was a fatty cerebral venous thrombosis that revealed hyperchylomicronemia that was complicated also by pancreatitis.
• During the hospitalization a cerebral tuberculosis abcess was diagnosed associated with nosocomial klebsiela pneumoniae sepsis and was succesfully treated.

## 1 Introduction

Hyperlipidemia is a lipid disorder that has many complications that could be life threatening situation such venous or arterial thrombosis, pancreatitis, and cardiovascular effect, and before searching a primary disorder, a secondary origin should be searched.

About 5% of hypertriglyceridaemia cases are of primary origin. The majority of these primary forms are incompletely characterised at the molecular level [1]. This statistical data suggests that several other gene variants may significantly influence the expression of hypertriglyceridaemia.

The prognosis of hypertriglyceridemia is determined either by an increased cardiovascular risk or by the occurrence of acute pancreatitis, mainly related to hypertriglyceridemia and also by how triglyceridemia is treated.

## 2 Case presentation

We report a case of a 4months-old patient with no medical history admitted in the emergency room for hypotonia appeared on the day of his admission without any traumatic context. The patient was hypotonic, and had a nystagmus, hemodynamically stable with heart rate at 135 beats per minute, blood pressure at 80/45 mmhg. oxygen saturation SpO 2% on room air was 96%, respiratory rate at 34 cpm, there were no signs of respiratory struggle the temperature was measured at 36.7° and capillary glycemia was at 1.07g/l. cranial perimeter at 47cm (+2DS) and his weight was at 6kg (-2DS).the patient had a moderate dehydration and had oliguria.

A full clinical examination revealed no pathological findings.

**Fig. 1:**
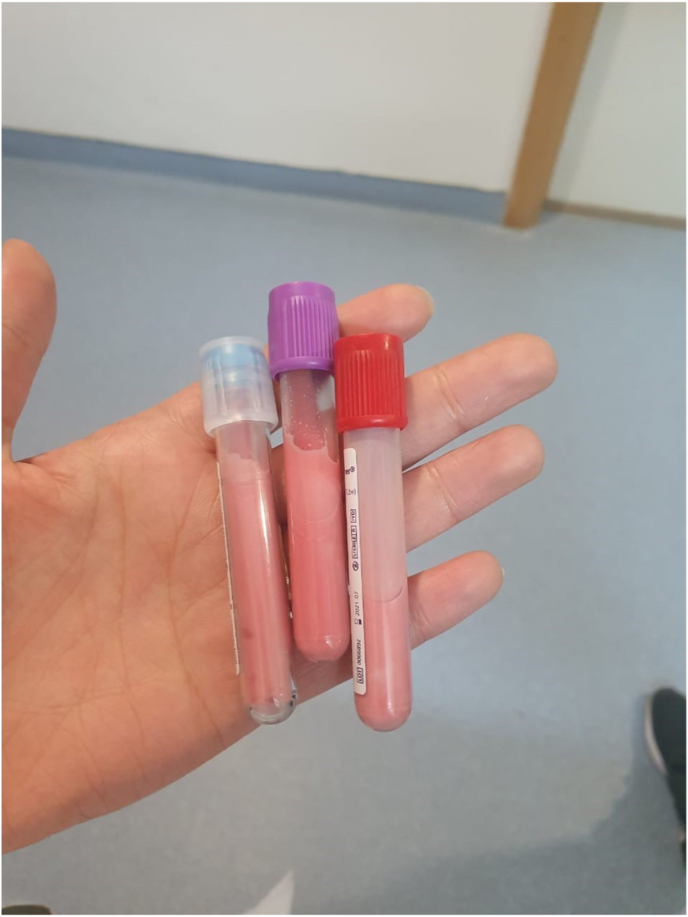
latescent macroscopic appearance of the patient blood sample.

The blood sample had a latescent macroscopic appearance (Fig. 1), the results are presented in Table 1.

**Table 1:** Biological findings.

Sodium	127mmol/l
Potassium	3.5mmol/l
calcium	115mg/l
albumin	56mg/ml
Cholesterol total	15.34 g/l
Cholesterol LDL	4.53 g/l
Triglycerides	HIGH (means that is more than 10 g/l)
Urea and creatinine	Couldn't be measured and another blood sample was made after correcting dehydration that showed Creatinine: 4.14 mg/l Urea: 0.17 mg/l
TP	100%
Platelets	147 000/mm3
White blood count	5690/mm3
Hemoglobin	17g/dl
Hematocrit	65%
Lymphocytes	4760/mm3
C-reactive protein	52.74 mg/l

**Fig. 2:**
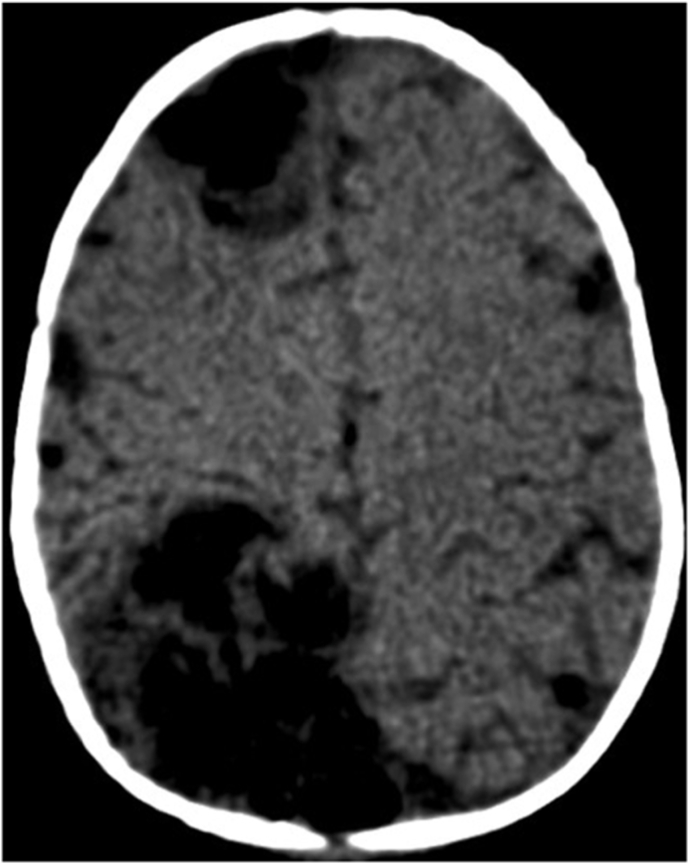
Head CT scan showing a right temporo-parietal hypodense area with suspicion of a cerebral venous thrombosis with no signs of intracranial hypertension.

An injected head CT SCAN was performed and showed a right temporo-parietal hypodense area with suspicion of a cerebral venous thrombosis with no signs of intracranial hypertension (Fig. 2)

Then the patient was admitted in the pediatric intensive care unit.

The patient was born from a first-degree consanguineous marriage, and never had any health problems such us convulsion episode, or neonatal infection. There was no familial medical history and the patient was the youngest of his 4 healthy siblings and was having maternal breastfeeding.

**Fig. 3:**
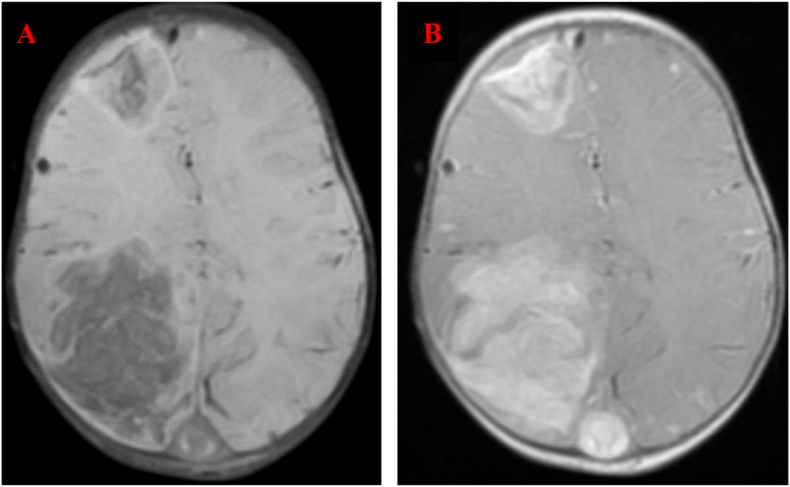
A) magnetic resonance imaging (MRI) T1-sequence showed right temporoparietal hypersignal B) MRI Fat-Saturation sequence showed a fading of the hypersignal.

**Fig. 4:**
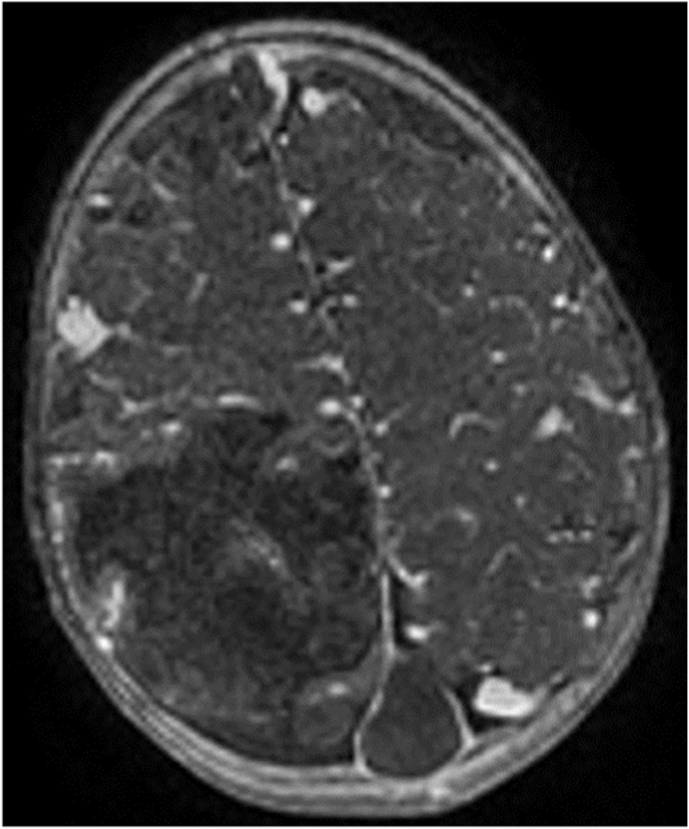
Lack of opacification of the dural venous sinuses in 3D FSPGR (fast spoiled gradient echo) MRI sequence.

A head magnetic resonance imaging (MRI) was performed right temporoparietal hypersignal in T1 sequence with fading on sequences with saturation of the fat signal (Fig. 3), with Lack of opacification of the dural venous sinuses in 3D FSPGR (fast spoiled gradient echo). (Fig. 4).

A spinal tap was performed and showed a normoglucorachic, hyperproteinorachic meningitis profile with 832 cells element.

So initially the patient was treated by antibiotherapy by ceftriaxone at the dose of 100mg/kg per day for his meningitis after correcting the dehydration. Curative dosing of Low molecular-weight heparin (LMWH) 1000UI/12H and 40mg per day of phenobarbital to prevent a convulsion episode.

The etiological diagnosis of familial hyperlipidemia was suspected due to the macroscopic aspect of the blood sample and the age of the patient as well as the lesions described on the MRI, but lipid tests in parents’ blood was normal and an electrophoresis of lipids was performed.

Another blood analysis was done containing: Complete lipid balance done in the standards (Bain Marie), hepatic, renal, thyroid functions that remains without abnormalities HIV test was negative, apolipoprotein 1, lipoprotein lipase activity and antibodies antilipopretein lipase was not available.

And to research other complications: lipasemia was tested and was 8 times the normal, so the patient was having a pancreatitis that we completed by abdominal CT scan after 48H that shows pancreatitis in stage A. also an echocardiography and fundus examination of both eyes that revealed no abnormalities.

**Fig. 5:**
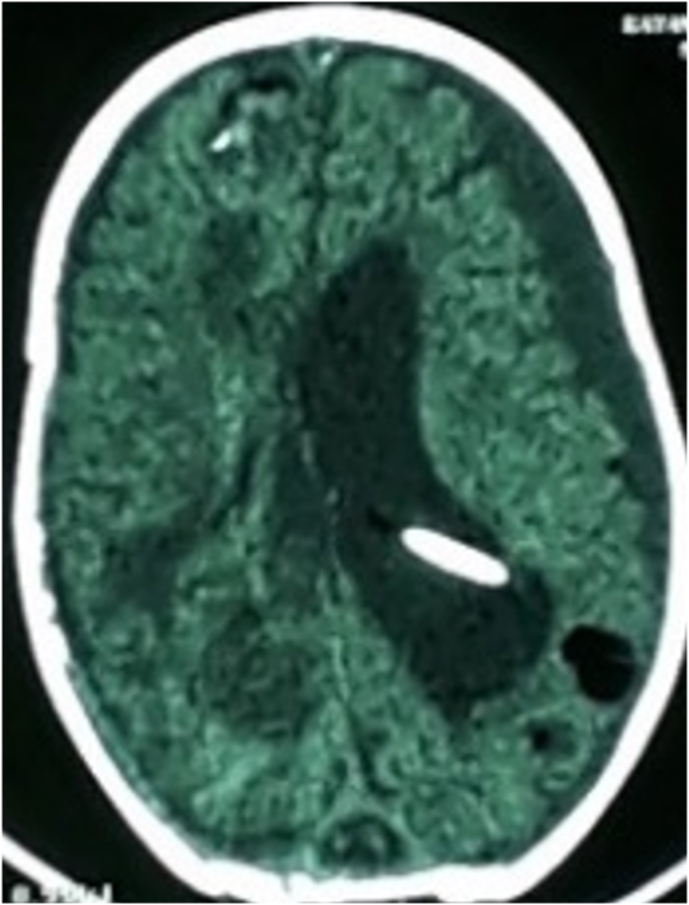
Head CT scan showing hydrocephalia with cerebral abscess.

The evolution after 7 days of hospitalization was marqued by the appearance of a fever at 39.1 with convulsive seizure disorder, so another head CT scan was performed that showed hydrocephalia with cerebral abscess of 3mm (Fig. 5). And a blood culture that revealed klebsiella pneumonia sensible to colistine and ciprofloxacin.

The neurosurgeons did an external ventricular by pass and drained the abscess that was sent to the anatomopathologist and the results was matching tuberculosis.

So, the patient was made under valproate of sodium 100 mg/12h phenobarbital 10 mg 4 pills per day colistine 40 mg/12h antituberculosis ERIP k4 ½ pill per day

With good clinical evolution, the patient didn't have any other convulsions episode and another spinal tap was performed which was negative.

When it comes to his lipid disorder a lipid electrophoresis of lipid showed a matched profile with chylomicronemia.

The patient was treated initially by low fat diet with not more than 25g of fat per day, fibrate can't be used as this age so we associated statin to the diet and the results were acceptable since triglyceride level becomes at 8.25 g/l and the patient was transferred to the pediatric department.

## 3 Discussion

Hyperlipidemias are linked to the risk of acute pancreatitis, venous and arteriel thrombosis and cardiovascular incidents which is life-threatening [1,2]. In our case the patient presented a cerebral venous thrombophlebitis and pancreatitis. Moderate primary hypertriglyceridemia are typically polygenic. They result from the accumulation of genes polymorphisms encoding for proteins involved in the triglycerides metabolism [3] and this was not our case since the parents blood tests for lipids was normal, this doesn't eliminate a genetic mutation but we couldn'*t*-test that since the genetic study was not available.

The diagnostic approach needs the knowledge of the metabolic basis of triglycerides and the etiopathogenesis. It consists, primarily, to dismiss a secondary origin before discussing a primary origin. Secondary origins are presented in Table 2 [4].

**Table 2:** Secondary causes.

Etiology	Tests
Diabetes	HBA1c, fasting blood glucose level
Metabolic syndrome	fasting blood glucose level, cholesterol HDL, blood pressure, outline of abdomen
Obesity	Body mass index
alcoholism	
Nephrotic syndrome	Edema, proteinuria
Renal injury	Creatinine
Hypothyroidism	TSH
Pregnancy	BHCG
Autoimmunity	Lipoprotein lipase antibodies
HIV infection	Serology

In our case the lipoprotein autoantibodies test was not available. When we can't find any secondary origin we procede to some other tests to search primary origin.

the appearance of the fasting serum is a systematic test preliminary to any other investigation that allows the biologist to orient the diagnosis of hypertriglyceridaemia. The appearance of fasting serum is normally clear. An opalescent or lactescent serum indicates the presence of presence of TGs,. The origin of the TGs is confirmed by the creaming test (endogenous or exogenous origin).

This routine check-up consists of measuring triglyceridemia and total cholesterol, on which the De Gennes classification is based, but also HDL cholesterol and LDL cholesterol. In our case the patient had latescent blood sample.

Hypertriglyceridaemia is preferentially accompanied by a change in distribution profile of LDL and shows an increase in the percentage of small and dense LDL [5].

LDL can thus be separated according to their density by ultracentrifugation into five major sub-fractions in a normolipidemic individual the LDL1 and LDL2 fractions represent the main fraction of LDL [6].

Familial chylomicronemia which a rare genetic disorder related to reduced or absent lipoprotein lipase activity. Its absence leads to hyperchylomicronemia after fasting or postprandial dosing because the LPL is responsible of the mediation of lipolysis of plasma chylomicron's triglyceride [7,8].

Primary chylomicronemia is the result of inactivating mutations in both alleles of LPL genes or other genes implicated in other proteins required for LPL activity [9,10] Apolipoprotein C-III is also implicated, its high levels present a risk factor for hypertriglyceridemia which is a the target in treatment by volanesorsen [11].

The management of this disease is based on low fat diet that should not be over than 25–30g per day [12], also statin [13,14], fibrate [15], omega 3 acid [16], heparin and insulin [17] can be used.

The SCARE guidelines were used in the writing of this paper [18].

## 4 Conclusion

Hyperchylomicronemia is a lipid disorder disease characterized by elevated lipid levels in the blood It consists, primarily, to dismiss a secondary origin before discussing a primary origin. It begins with the lipid profile before resorting to lipoprotein electrophoresis and post heparin LPL activity measurement. Adequate exploration permits to obtain the optimum care to avoid any complications such us thrombosis, pancreatitis, and cardiovascular events.

## Ethical approval

The ethical committee approval was not required give the article type case report. However, the written consent to publish the clinical data of the patients was given and is available to check by the handling editor if needed.

## Sources of funding

This research did not receive any specific grant from funding agencies in the public, commercial, or not-for-profit sectors.

## Funding

This research did not receive any specific grant from funding agencies in the public, commercial, or not-for-profit sectors.

## Registration of research studies

This is not an original research project involving human participants in an interventional or an observational study but a case report. This registration is was not required.

## Guarantor

Ounci Es-Saad

## Consent

The consent was obtained from the children's holders.

## Provenance and peer review

Not commissioned, externally peer reviewed.

## Consent for publication

Written informed Consent was obtained from the child's parents for publication of this case report and accompanying images.

The authors state that they have no conflicts of interest for this report.

This research did not receive any specific grant from funding agencies in the public, commercial, or not-for-profit sectors.

The ethical committee approval was not required give the article type (case report).However, the written consent to publish the clinical data of the patients was given and is available to check by the handling editor if needed.

Written informed Consent was obtained from the child's parents for publication of this case report and accompanying images.

Ounci Es-saad: study concept or design, data collection, data analysis or interpretation, writing the paper.

Oussama Lamzouri: data analysis or interpretation, writing the paper.

Amine Bouchlarhem: : data analysis or interpretation, writing the paper.

Aabdi Mohammed: : data analysis or interpretation.

Leila Haddar: data collection.

Hamza mimouni: data collection.

Kaoutar zerouati: data collection.

Houssam bkiyar: supervision and data validation.

Brahim Housni: supervision and data validation.

This is not an original research project involving human participants in an interventional or an observational study but a case report. This registration is was not required.

## Declaration of competing interest

The authors state that they have no conflicts of interest for this report.
